# Non-Small Cell Lung Cancer (NSCLC) in Young Adults, Age < 50, Is Associated with Late Stage at Presentation and a Very Poor Prognosis in Patients That Do Not Have a Targeted Therapy Option: A Real-World Study

**DOI:** 10.3390/cancers14246056

**Published:** 2022-12-09

**Authors:** Daniel Johnathan Hughes, Matthaios Kapiris, Andreja Podvez Nevajda, Harriet McGrath, Chara Stavraka, Shahreen Ahmad, Benjamin Taylor, Gary J. R. Cook, Sharmistha Ghosh, Debra Josephs, Elias Pintus, Spyridon Gennatas, Andrea Bille, Kimuli Ryanna, George Santis, Ana Montes, Mieke Van Hemelrijck, Eleni Karapanagiotou, Daniel Smith, James Spicer, Alexandros Georgiou

**Affiliations:** 1 Department of Cancer Imaging, School of Biomedical Engineering and Imaging Sciences, King’s College London, Lambeth Wing, St Thomas’ Hospital, Westminster Bridge Road, London SE1 7EH, UK; 2 Cancer Centre at Guy’s, Guy’s and St Thomas’ NHS Foundation Trust, Great Maze Pond, London SE1 9RT, UK; 3 School of Cancer and Pharmaceutical Sciences, King’s College London, Guy’s Campus, Great Maze Pond, London SE1 1UL, UK; 4 King’s College Hospital NHS Foundation Trust, King’s College Hospital, Denmark Hill, London SE5 9RS, UK; 5 Lewisham and Greenwich NHS Trust, University Hospital Lewisham, High Street, Lewisham, London SE13 6LH, UK

**Keywords:** non-small cell lung cancer, young adults, molecular targeted therapies, precision medicine, genetic predictive testing

## Abstract

**Simple Summary:**

Non-small cell lung cancer in young adults spans all ethnic backgrounds and is associated with advanced disease and poor outcomes. Identification of genetic changes that are associated with disease and can subsequently be targeted with specific therapies is associated with improved survival. As such, comprehensive molecular testing is recommended in all advanced young adults with non-small cell lung cancer.

**Abstract:**

(1) Background: Non-small cell lung cancer (NSCLC) in young patients is uncommon. Real-world evidence on the outcomes of these patients is limited. (2) Methods: We conducted a retrospective cohort study of young NSCLC patients, age < 50 years at diagnosis, who were treated between 2011–2020 in South-East-London cancer centres. Clinicopathological characteristics, treatment and outcomes were analysed. (3) Results: Of 248 NSCLC patients, median age was 46 years, 50% (*n* = 125) female, 58% (*n* = 145) white, 18% (*n* = 45) black and 4% (*n* = 10) Asian ethnicity. Amongst patients with a documented smoking history, 30% (*n* = 64) were never-smokers. Most patients had adenocarcinoma (77%, *n* = 191) and presented with metastatic disease (67%, *n* = 166). Only 31% (*n* = 76) had treatment with curative intent. In patients who presented or developed metastatic non-squamous NSCLC (*n* = 179), *EGFR* mutation status was known in 88% (*n* = 157) and mutation present in 19% (*n* = 34), *ALK* was known in 66% (*n* = 118) with a translocation in 10% (*n* = 18), *ROS1* status was known in 57% (*n* = 102) with a translocation in 4% (*n* = 8), and *KRAS* status was known in 66% (*n* = 119) with a mutation in 12% (*n* = 22). Overall, 76% (*n* = 152) patients with metastatic NSCLC received first-line systemic anti-cancer therapy. Median overall survival in metastatic NSCLC was 9.0 months (95% CI 6.5–11.6 months), with superior median overall survival in those with a targeted therapy option (28.7 months) compared to those without (6.6 months; *p* < 0.001). (4) Conclusion: Young patients contribute a significant proportion of those presenting with lung cancer. They present with advanced stage at diagnosis and have a poor prognosis. Identification of a targeted therapy option is associated with improved survival. However, most patients do not have a known genomic driver, which is in part due to limited testing, particularly in the early years of this study period. These findings highlight the particular importance of rapid-turnaround comprehensive genomic profiling in this age group and the need to identify strategies to facilitate earlier diagnosis in young NSCLC patients.

## 1. Introduction

Lung cancer is the leading cause of cancer-related deaths globally, with over 2.2 million new cases and 1.8 million deaths in 2020 alone [1]. Despite advances in the past two decades, the 5-year overall survival in the United Kingdom (UK) remains poor, being 3% in those with advanced disease [2]. Non-small cell lung cancer (NSCLC) accounts for up to 90% of all lung cancer cases in the UK, with a median age at diagnosis of 72 years [3,4]. Lung cancer in young patients, defined in this study as age < 50 years at the time of diagnosis, remains relatively uncommon making up just 2.5% of all cases in the 2020 National Lung Cancer Audit [4]. The rates of tobacco smoking in younger adults in the UK is decreasing. At the same time, the incidence of NSCLC in young patients remains stable between 5 and 6 per 100,000 since the early 2000s, thereby suggesting a role for alternative aetiology [5]. Of particular interest in urban populations, recent research highlighted the role of air pollution, with increasing concentrations of particulate matter associated with increased risk of mutant epidermal growth factor receptor (*EGFR*) NSCLC, particularly in never-smokers [6]. Several studies have highlighted differences in the characteristics of young NSCLC patients when compared to the general population. Younger patients have a higher proportion of female patients, lower smoking rates and an increased likelihood of presentation at an advanced stage [7,8,9,10]. Notably there appears to be a lack of consensus of how to define young onset lung cancer with variable definitions across studies, ranging from age at diagnosis of <35 to <50 years [7,8,9,10].

NSCLC is a heterogeneous disease with an increasing number of genetic aberrations now recognised to predict disease biology, treatment, and outcomes. Over the past two decades, treatment with agents targeting *EGFR* gene mutations, present in approximately 10% of NSCLC cases in the UK, anaplastic lymphoma kinase (*ALK*) and ROS proto-oncogene 1 (*ROS1)* translocations have led to an improved survival of these patients in the metastatic setting [7,11]. These genetic aberrations are known to be more common in young onset NSCLC.

There are conflicting reports relating to the outcomes of young NSCLC patients. A large US cohort described poor NSCLC survival outcomes, despite higher incidence of targetable mutations, suggestive of an aggressive biology [10]. In contrast, studies with predominant Asian population report a better survival when compared to older NSCLC patients [8,12].

We aimed to profile the clinicopathological characteristics and treatment outcomes of patients with young age onset NSCLC (age < 50 years) in the real-world setting in a high-volume tertiary South East London comprehensive cancer centre.

## 2. Materials and Methods

### 2.1. Data Collection

Patients aged 18–50 years with a diagnosis of ICD10 C34 (malignant neoplasm of bronchia and lung) who were treated at King’s Health Partners Comprehensive Cancer Centre between March 2011 and March 2020 were identified using electronic medical records. Lung cancer diagnosis date was defined as the date of histopathological or cytological diagnosis. Patients who had received treatment in other centres, in whom treatment outcomes were not available for extraction were excluded.

Electronic medical records of included patients were reviewed for patient demographics including age, sex, ethnicity, smoking history, date of diagnosis, histology, molecular analysis, staging, performance status, treatment history, and survival data. Routine molecular characterisation of patients’ tumours with stage IV disease evolved during 2011–2020, as did the availability of targeted therapies. We collected data for all the genes/proteins that were tested as part of standard of care during the data collection phase at Q1/2 of 2020. This included *EGFR*, *BRAF, KRAS* mutation status and *ALK*, *ROS1* testing with either immunohistochemistry and/or fluorescence in situ hybridization (FISH). The variable rate of data completion reflects the fact that some genes, e.g., *EGFR,* were tested from early 2010s, whereas other targets, e.g., *ROS-1* testing, were adopted in standard of care testing later on. At the time of data collection cut off *RET*, *NTRK* 1–3 rearrangements, *MET* exon 14 skipping mutations were not routinely tested in our centre and therefore were not included in our analysis.

The study underwent institutional review (approval no. 11127), with research ethics committee approval (ref: 18/NW/0297) within the previously described Guy’s Cancer Cohort framework [13].

### 2.2. Statistics

Patients were observed until May 2020 or to date of death, whichever was earliest. Data are presented in subgroups defined by age 18–30, 31–40, and 41–50 years. Categorical data were compared with χ2 test whilst continuous variables were compared using independent *t*-test. Composite variable for targetable genomic alterations (TKI-option) was used if at least one actionable aberration in *EGFR*, *ALK* or *ROS1* was present. Overall survival was defined as time from date of diagnosis of metastatic disease (histopathological or radiological) to date of death from any cause and patients alive at data cut-off were censored at time of last known follow-up. Survival was analysed using the Kaplan–Meier method with log-rank. Multivariate Cox models were used to determine the effect of age on survival, controlling for sex, smoking history, ethnicity, number of metastatic sites, presence of brain metastases, histological subtype, and presence of targetable genomic alteration. *p* values are two-sided with significance as α = 0.05. Data were analysed using GraphPad Prism v9.4.0 for macOS (GraphPad Software, San Diego, CA, USA) and IBM SPSS statistics v27.0.1.0 for macOS (IBM Corp., Armonk, NY, USA).

## 3. Results

### 3.1. Clinical Characteristics

A total 368 patients (of 5624 total; 6.5%) aged 18–50 years were identified between March 2011 to March 2020. Sixty-seven patients were excluded due to insufficient data, as they were treated in centres that were not included in this study.

Of 301 patients, 248 (82%) had NSCLC, 27 (9%) SCLC and 26 (9%) carcinoid histology (Supplementary Table S1). The number of new NSCLC diagnoses annually in patients aged 18–50 years appeared stable over the study period (median = 27 patients annually, range 23–32).

The demographics and clinicopathological characteristics of NSCLC patients are summarised in Table 1. The majority of NSCLC patients (*n* = 248) had adenocarcinoma (*n* = 191, 77%), followed by 33 (13%) squamous cell carcinoma, 21 (8%) NSCLC-not otherwise specified (NOS), and 3 (1%) large cell carcinoma. The median age was 46 years, with 9 (4%) patients in the 18–30 years, 52 (21%) in the 31–40 years, and 187 (75%) in the 41–50 years age groups. Patients were equally distributed in terms of sex with a higher-than-expected proportion of females to males (1:1) compared to the general UK NSCLC population (1:1.4) [3]. The majority presented with advanced disease (stage III, *n* = 43 (17%); stage IV, *n* = 166 (67%)). Of all NSCLC patients with an available smoking history, 210 (30%) were never-smokers.

Our cohort of young NSCLC patients has a multi-ethnic profile representative of the local demographics of South-East London. The majority (*n* = 145, 58%) were white, 45 (18%) were black and 10 (4%) Asian. Ethnicity was recorded as mixed, other or unknown in the remaining cases (*n* = 48, 19%).

Overall, 199 (80%) NSCLC patients were diagnosed with or developed metastatic disease during their disease course (Table 2). Brain metastases were common, detected in 70 (35%) of metastatic NSCLC patients at any time-point. The majority of patients presented with good Eastern Cooperative Oncology Group (ECOG) performance status (PS) 0–1 (71%). However, the number of patients with PS ≥ 2 (*n* = 34, 17%) at presentation is higher than expected given that this was a cohort of young patients. Characteristics also differed between ethnic groups. Never-smoker status was higher in black and Asian patients with NSCLC (37% and 40%, respectively) compared to white patients (23%).

### 3.2. Molecular Diagnostics in Metastatic NSCLC Patients

Of all metastatic NSCLC patients (*n* = 199), the majority were non-squamous histological subtype (*n* = 179). Of the non-squamous metastatic NSCLC patients, 160 (89%) underwent genomic analysis. Most patients who did not undergo genomic analysis (*n* = 13, 7%) did not receive first-line systemic anti-cancer therapy (SACT), due to poor performance status at presentation associated with a very short survival of ≤30 days from diagnosis in 10/13 cases.

**Figure 1:**
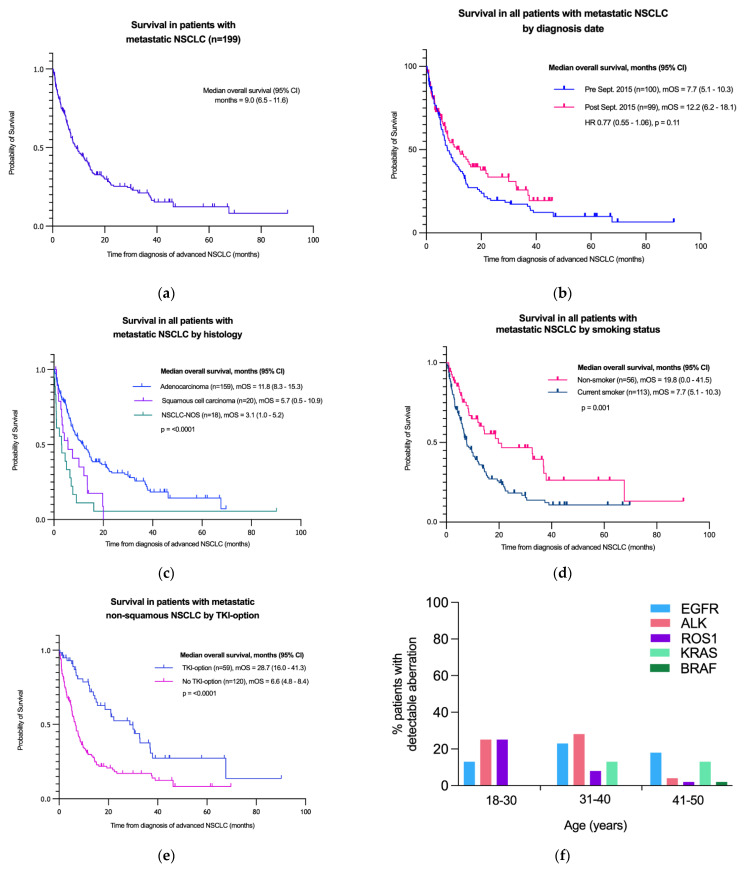
Survival analyses of (**a**) all metastatic NSCLC, (**b**) by diagnosis date, (**c**) by histopathology, (**d**) by smoking status and (**e**) by availability of TKI-option with (**f**) frequency of driver mutations by age group.

The *EGFR* mutation status was known in 157 (88%) patients. *ALK* status was known in 118 (66%), *ROS1* in 102 (57%), *KRAS* in 119 (66%) and *BRAF* in 53 (30%) patients. The variable testing completion rates reflect the sequential expansion of molecular testing in NSCLC during 2011–2020 in the UK. Patients who were diagnosed during earlier years included in this study had a smaller molecular diagnostic panel. Overall, 34 (19%) were found to have an *EGFR* mutation, 18 (10%) *ALK* translocation, 8 (4%) *ROS1* translocation, 22 (12%) *KRAS* and 2 (1%) *BRAF* mutations. The frequency of genomic aberrations varied between age sub-groups (Table 2, Figure 1), with *ALK* and *ROS1* translocations being more common in the youngest patients (e.g., *ALK* translocation noted in 25%, 28% and 4% of the 18–30, 31–40 and 41–50 years groups, respectively). *EGFR* mutations were seen across all age sub-groups, whereas *KRAS* mutations were more common in the older (41–50 years) subgroup.

When considering results per ethnic subgroup, activating *EGFR* mutations were detected in 44%, 28% and 14% of non-squamous metastatic NSCLC tumours in Asian, black and white patients, respectively (*p* = 0.001) and *ALK* or *ROS1* re-arrangement were detected in 22%, 3% and 17%, respectively (*p* = 0.2).

Fifty-six (28%) metastatic NSCLC patients were never-smokers (Supplementary Table S2). Never-smokers were younger (median age 42 years) and predominantly female (*n* = 41, 73%) with adenocarcinoma histology (*n* = 48, 86%). Genetic aberrations were more common in never-smokers accounting for 57% of those with an *EGFR* mutation, 61% of *ALK* translocation and 56% of *ROS1* translocation. Of those with a *KRAS* mutation, only 2 (9%) were never-smokers.

Programmed death-ligand 1 (PD-L1) status was known in 82 (41%) cases. Out of patients with known PD-L1, 33 (39%) had PD-L1 expression < 1%, 18 (21%) PD-L1 of 1–49% and 33 (39%) had PDL1 ≥ 50%.

### 3.3. Treatment and Survival

Of all young patients with NSCLC (*n* = 248), only 30% had treatment with curative intent. Thirty-two (13%) young patients with NSCLC were treated with (chemo)radiotherapy, of which 28 (88%) received curative intent radiotherapy dose. However, locoregional (*n* = 4, 13%) and distant (*n* = 14, 44%) relapse post (chemo)radiotherapy was common. Median overall survival (mOS) from diagnosis was 14.6 months (95% CI 2.9–26.4 m).

Forty-four (17%) young patients with NSCLC received curative intent surgery, of which 42 (95%) had an R0 resection. Locoregional (*n* = 1, 2%) and distant (*n* = 8, 18%) relapse post-surgery was less common and mOS in this group was not reached.

**Figure 2:**
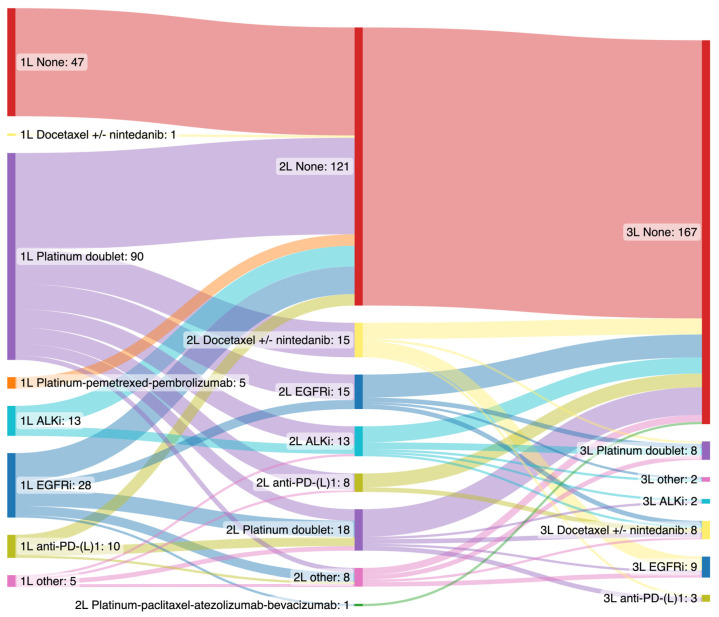
Systemic anti-cancer therapy (SACT) frequencies of all young metastatic NSCLC patients (*n* = 199) by sequential lines of therapy (1L—first line; 2L—second line; 3L—third line; ALKi—ALK tyrosine kinase inhibitor; anti-PD-(L)1—anti-programmed cell death protein-1/programmed death-ligand 1; EGFRi—EGFR tyrosine kinase inhibitor).

A total of 152 of 199 (76%) patients with metastatic NSCLC received first-line systemic anti-cancer therapy (SACT). The most commonly administered first-line SACT was platinum-doublet chemotherapy (*n* = 90, 45%) (Figure 2). Almost half (49%) of those who had received first-line SACT did not have second-line and 58% of those who had received second-line therapy did not proceed to third-line. Across all lines of therapy, there were 22 (14%) clinical trial participants. A significant proportion of patients (*n* = 47, 24%) did not receive SACT due to poor performance status (PS ≥ 2) with a majority of these patients (*n* = 34, 72%) having a poor prognosis with overall survival ≤ 90 days.

Palliative radiotherapy was relatively common, 70 (35%) metastatic NSCLC patients, while a high proportion had whole-brain (*n* = 44, 22%) and stereotactic (*n* = 11, 6%) radiotherapy for brain metastases (Table 3).

Overall, the mOS of all patients with metastatic NSCLC was 9.0 months (95% CI 6.5–11.6 months). Patients with squamous cell carcinoma had a poorer mOS of 5.7 m (95% CI 0.5–10.9 m) compared to 11.8 m (95% CI 8.3–15.3 m) in those with adenocarcinoma (Figure 1). There was a trend towards improved mOS of all metastatic NSCLC patients diagnosed after (12.2 m) and before (7.7 m) the study mid-point of September 2015 (non-adjusted, see demographics Supplementary Table S3, Figure 1).

Median OS varied at 5.4 m (95% CI 0.0–14.2 m) in 18–30 years, 15.2 m (95% CI 7.4–23.0 m) in 31–40 years, and 7.8 m (95% CI 5.6–9.9 m) in 41–50 years age groups, but with no statistical significance (*p* = 0.378). There was no statistical difference (*p* = 0.095) between mOS of females (11.3 m; 95% CI 7.0–15.6) and males (7.7 m; 95% CI 5.3–10.1).

Never-smokers had an improved mOS at 19.7 m (95% CI 0.0–41.5) compared to 7.7 m (95% CI 5.1–10.3) for those with a known smoking history (*p* = 0.001). We further analysed mOS in metastatic non-squamous NSCLC patients according to whether or not they had a targeted tyrosine kinase inhibitor (TKI) therapy option (i.e., known *EGFR* kinase domain mutation or *ALK*/*ROS1* translocations). We found those who had an anti-EGFR/ALK or ROS1 TKI-option had a statistically significant (*p* < 0.001, HR 0.41, 95%CI 0.29–0.58) longer mOS of 28.7 m (95% CI 16.0–41.3) vs. 6.6 m (95% CI 4.8–8.4) in those without a targeted therapy-option (Figure 1). This remained statistically significant in multivariate analysis (HR 0.42, 95% CI 0.24–0.69, *p* < 0.001) (Table 4).

We also analysed our treatment and outcome results by ethnic subgroups. A similar proportion of black (76%) and white (75%) patients and 100% of Asian patients were treated with first-line SACT. The mOS of black and white patients was poor (12.0 months, 95% CI 7.1–16.8 m; 9.0 months, 95% CI 6.9–11.2 m, respectively). In Asian patients, mOS was 30.5 months (95% CI 10.4–50.6 m), (across all groups, *p* = 0.38).

Multivariate analysis also indicated a survival benefit in those with only one site of metastatic disease (HR 0.38, 95% CI 0.24–0.62, *p* < 0.001). A survival disadvantage was noted in those with NSCLC-NOS (HR 2.28, 95% CI 1.26–3.94, *p* = 0.004) and large cell carcinoma (HR 13.02, 95% CI 1.92–52.6, *p* = 0.001) histological subtypes.

## 4. Discussion

This real world, London (UK) based study provides further evidence that young lung cancer patients contribute a significant number of patients, herein accounting for 6.5% of all our lung cancer patients.

Compared to an average age-unrestricted NSCLC cohort, our younger cohort had a higher-than-expected proportion of female and never smoker patients [7,8,9]. The ethnic diversity of our cohort is reflective of the diverse London population but also proves that NSCLC in young patients spans all ethnic groups. Historically prospective interventional studies have small numbers of black patients or have grouped different ethnic backgrounds as one cohort (e.g., Asian vs. non-Asian) [14,15]. Given the unique characteristics of black vs. white vs. Asian patients, it is important that future studies focus on recruitment of patients from all ethnic backgrounds and clearly report the full ethnic breakdown of the trial participants.

Consistent with other studies and UK Office of National Statistics data, young NSCLC patients in our cohort presented with advanced stage III/IV (84%) disease [8,10,12]. This may suggest delayed presentation due to limited symptoms in younger adults with a higher physiological reserve, and possibly a lower index of suspicion amongst clinicians. More work is required to promote awareness of lung cancer in younger adults including in non-smokers. The roll out of lung cancer screening programmes has the potential to detect NSCLC at an earlier stage. However, in most countries these will exclude patients <50 years old and/or non-smokers. Therefore, screening will not facilitate earlier diagnosis in younger patients. More dedicated research is required to understand risk factors that contribute to the development of NSCLC, particularly as in many patients tobacco smoking exposure was unlikely to be a contributing factor. Qualitative research is also required to understand patterns of presentation in this population in order to develop dedicated strategies that can facilitate earlier diagnosis.

Tumour biology in young patients is also a likely contributing factor to the high proportion presenting with advanced disease. Compared to older patients, young adults have better vascular supply and, as such, access to oxygen and glucose, resulting in greater tumourigenesis and larger tumours [16]. This in turn may result in greater tumour hypoxia, increased hypoxia-inducible factor 1 (HIF-1) and associated epithelial-mesenchymal transition, a pre-requisite for metastasis [17]. Oxidative stress as a result of hypoxia-induced reactive oxygen species may also partially explain the increased incidence of genetic alterations in the young NSCLC population [18]. Ongoing translational work evaluating cancer biology and evolution, such as the TRACERx (NCT01888601) and PEACE (NCT03004755) studies, should hopefully address such questions, however, it is important that young NSCLC patients are actively recruited to such studies, as well as, local biobanks [19].

Our patients had predominantly adenocarcinomas (80% of all NSCLC), which may be partly explained by lower smoking rates and exposure in this cohort. Several other studies conducted in national cohorts in the US and Japan have demonstrated similarly high rates of adenocarcinoma in the young NSCLC population (77–87%) [7,9,12]. The largest UK data set reported adenocarcinoma in 48% of those aged 18–39 years but there was a high proportion of missing data [3].

In non-squamous NSCLC patients, we found higher rates of targetable mutations in *EGFR*, *ALK*, and *ROS1* (19%, 10%, 4%) than that expected in a typical NSCLC age-unrestricted UK population [2,3]. Specifically, *ALK* translocations were detected more frequently in the youngest patients, age ≤ 40. This is an increasingly reported finding in the young NSCLC population of other primarily white populations [7].

In the current literature there is conflicting evidence of whether younger age at diagnosis is associated with improved survival compared to older [3,7,8,9,10,12]. It is not possible to directly compare these real-world studies with significant differences in population demographics and non-standardised definition of young NSCLC patient. In our study, prognosis in patients aged <50 years with metastatic NSCLC was very poor but notably improved in the 59 patients (33%) who had a targeted therapy (anti-EGFR/ALK/ROS1 TKI) option (mOS 28.7 vs. 6.6 m). In addition, we demonstrated a trend towards an improvement in survival for those diagnosed post vs. pre the study midpoint of September 2015 (12.2 vs. 7.7 months; *p* = 0.11). This observation is also likely to reflect our increasing rates of genotyping and access to targeted therapies in the latter years of this study.

Overall, our results strongly support the paramount importance of rapid-turnaround comprehensive genomic profiling and personalised treatment strategy in advanced young NSCLC patients. Over the past 2 years our standard of care tissue molecular diagnostic panel has expanded. Next-generation sequencing (NGS) platforms are now routinely used allowing for the concurrent sequencing of multiple genes. Moreover, improved RNA-sequencing analysis is increasingly used for the parallel analysis of gene expression and fusion variants. At the same time newer treatments targeting rare resistant exon 20 *EGFR* mutation, *MET exon 14* ‘skipping’ mutations as well as *RET* and *NTRK1-3* fusion variants have recently been licensed. Targeting *ERBB2* mutations and *NRG1* gene fusions are also in late development. These advances are likely to further increase the number of patients with targeted therapy options and overall outcomes of young NSCLC patients in the coming years. Given the extremely poor OS of young fit patients without targeted therapy options we also advocate for concurrent testing using blood cell free (cf) DNA at diagnosis in all NSCLC patients age <50 years old, as complementary to tissue NGS for biomarker evaluation. Molecular testing using validated cfDNA platforms show high concordance with tissue-based testing in NSCLC, with a faster turnaround time as well as detection of driver alterations in patients with negative or insufficient tissue results [19,20,21]. Our results suggest that the young NSCLC population is a suitable cohort for prospective studies evaluating the real-life cost-effectiveness of commercially available and academic cfDNA platforms. The dedicated study of the relationship between comprehensive genetic signatures and possible environmental risk factors in young adults with NSCLC may provide information that can then be used to derive strategies for earlier diagnosis and newer therapies.

There are some limitations to our study. Data analysis was performed retrospectively and collected from a limited geographical catchment area. Additionally, data on risk factors beyond smoking status including occupation, asbestos exposure, personal history of respiratory comorbidities and family history of lung cancer were incomplete. Genotyping was performed as per standard of care at diagnosis, with which access to and the testing platform used has evolved over the studied time period and therefore was not consistent between all patients. We were unable to investigate the role of immunotherapy and chemoimmunotherapy as these therapies were not available for the majority of the duration of our study. Additionally, PD-L1 testing was not available for the majority of patients in this cohort, the assay used over the study period changed, and very few patients received an anti-PD-1/PD-L1 therapy with a short follow-up period in those that did. The role of immune checkpoint inhibition in a young adult NSCLC population is an important gap in the literature that should be explored in future prospective studies. In this retrospective analysis, data on quality of life and the impact of psychosocial aspects in our young NSCLC population was not available. A diagnosis of NSCLC in young adults can be associated with psychological upheaval, lack of acceptance and even stigma, with significant effect on family and professional life. An observational cohort with the collection of such data would be highly valuable.

## 5. Conclusions

This study provides important insight into the characteristics of NSCLC in young patients, <50 years old. These patients span all ethnic backgrounds with a higher proportion of non-smoker females when compared to a typical age-unrestricted UK NSCLC cohort. Unfortunately, these patients more frequently present with advanced stage at diagnosis and have a very poor prognosis despite higher-than-average fitness levels. Patients with advanced non-squamous NSCLC who had a known targeted therapy option had a significantly improved survival compared to those without, emphasising the importance of rapid-turnaround, extended genomic analysis irrespective of smoking status. With a continuously evolving treatment landscape, further multi-centre cohorts are needed to better define the clinical and biological characteristics of this unique patient population and inform clinical practice.

## Figures and Tables

**Table 1:** Clinical characteristics of all NSCLC patients < 50 years (*n* = 248).

Clinical Characteristic	Total *n* = 248
**Age at diagnosis, *n*** (%)	
18–30	9 (4)
31–40	52 (21)
41–50	187 (75)
Mean (yrs)	44
Median (yrs)	46
Range (yrs)	26–50
**Sex, *n*** (%)	
Female	125 (50)
Male	123 (50)
**Smoking history, *n*** (%)	
Never smoker	64 (26)
Ex-smoker	51 (21)
Smoker	95 (38)
Unknown	38 (15)
**Ethnic background, *n*** (%)	
White (British, Irish, other)	145 (58)
Black (African, Caribbean, British)	45 (18)
Asian (Asian, British)	10 (4)
Mixed	3 (1)
Other	7 (3)
Not stated	38 (15)
**Stage at diagnosis, *n*** (%)	
I	24 (10)
II	15 (6)
III	43 (17)
IV	166 (67)
**Histopathology, *n*** (%)	
Adenocarcinoma	191 (77)
Squamous cell carcinoma	33 (13)
Large cell carcinoma	3 (1)
NSCLC-NOS	21 (8)

NOS—not otherwise specified.

**Table 2:** Clinical characteristics of all metastatic NSCLC patients (*n* = 199; at diagnosis or developed metastatic disease) by age group.

Clinical Characteristic	All, *n* (% of Total)	18–30 Years	31–40 Years	41–50 Years
**Age at diagnosis, *n*** (%)	**199 (100)**	9 (5)	44 (22)	146 (73)
Mean (yrs)	**44**			
Median (yrs)	**45**			
Range (yrs)	**26–50**			
**Sex, *n*** (%)				
Female	**97 (49)**	5 (56)	23 (52)	69 (47)
Male	**102 (51)**	4 (44)	21 (48)	77 (53)
**Smoking history, *n*** (%)				
Never smoker	**56 (28)**	5 (56)	20 (45)	31 (21)
Ex-smoker	**38 (19)**	1 (11)	11 (25)	26 (18)
Smoker	**75 (38)**	1 (11)	11 (25)	63 (43)
Unknown	**30 (15)**	2 (22)	2 (5)	26 (18)
**Ethnic background, *n*** (%)				
White (British, Irish, other)	**109 (55)**	7 (78)	24 (55)	78 (53)
Black (African, Caribbean, British)	**41 (21)**	0 (0)	6 (14)	35 (24)
Asian (Asian, British)	**10 (5)**	2 (22)	5 (11)	3 (2)
Mixed	**3 (2)**	0 (0)	2 (5)	1 (1)
Other	**4 (2)**	0 (0)	1 (2)	3 (2)
Not stated	**32 (16)**	0 (0)	6 (14)	26 (18)
**Stage at diagnosis, *n*** (%)				
I	**6 (3)**	0 (0)	0 (0)	6 (4)
II	**2 (1)**	0 (0)	0 (0)	2 (1)
III	**25 (13)**	2 (22)	2 (5)	21 (14)
IV	**166 (83)**	7 (78)	42 (95)	117 (80)
Number of metastatic sites *, *n* (%)				
1	**69 (35)**	1 (11)	11(25)	57 (39)
2	**73 (37)**	4 (44)	18 (41)	51 (35)
>3	**57 (29)**	4 (44)	15 (34)	38 (26)
Metastatic sites *, *n* (%)				
Intrathoracic	**98 (49)**	6 (67)	31 (70)	61 (42)
Extrathoracic				
Brain	**65 (33)**	3 (33)	12 (28)	50 (34)
Bone	**82 (41)**	6 (67)	14 (32)	62 (42)
Liver	**37 (19)**	3 (33)	10 (23)	24 (16)
Adrenal	**35 (18)**	1 (11)	6 (14)	28 (19)
Distal lymph nodes	**34 (17)**	4 (44)	7 (16)	23 (16)
other	**21 (11)**	2 (22)	3 (7)	16 (11)
**Performance status at** **diagnosis, *n*** (%)				
0	**39 (20)**	0 (0)	10 (23)	29 (20)
1	**101 (51)**	9 (100)	26 (59)	66 (45)
2	**18 (9)**	0 (0)	2 (5)	16 (11)
3	**12 (6)**	0 (0)	2 (5)	10 (7)
4	**4 (2)**	0 (0)	0 (0)	4 (3)
unknown	**25 (13)**	0 (0)	4 (9)	21 (14)
**Histopathology, *n*** (%)				
Adenocarcinoma	**159 (80)**	8 (89)	38 (86)	113 (77)
Squamous cell carcinoma	**20 (10)**	1 (11)	4 (9)	15 (10)
Large cell carcinoma	**2 (1)**	0 (0)	1 (2)	1 (1)
NSCLC-NOS	**18 (9)**	0 (0)	1 (2)	17 (12)
**PD-L1 score, *n*** (%)				
<1	**32 (16)**	0 (0)	12 (27)	20 (14)
1–49	**18 (9)**	0 (0)	5 (11)	13 (9)
≥50	**32 (16)**	3 (33)	11 (25)	18 (12)
Unknown	**117 (59)**	6 (67)	16 (36)	95 (65)
***EGFR* status in** **Non-squamous, *n*** (%)				
Mutant	**34 (19)**	1 (13)	9 (23)	24 (18)
Wild type	**123 (69)**	7 (88)	28 (70)	88 (67)
Unknown	**22 (12)**	0 (0)	3 (8)	19 (15)
***ALK* status in** **Non-squamous, *n*** (%)				
Rearranged	**18 (10)**	2 (25)	11 (28)	5 (4)
Not rearranged	**100 (56)**	4 (50)	24 (60)	72 (55)
Unknown	**61 (34)**	2 (25)	5 (13)	54 (41)
***ROS1* status in** **Non-squamous, *n*** (%)				
Rearranged	**8 (4)**	2 (25)	3 (8)	3 (2)
Not rearranged	**94 (53)**	4 (50)	28 (70)	62 (47)
Unknown	**77 (43)**	2 (25)	9 (23)	66 (50)
***KRAS* status in** **Non-squamous, *n*** (%)				
Mutant	**22 (12)**	0 (0)	5 (13)	17 (13)
Wild type	**97 (54)**	4 (50)	24 (60)	69 (53)
Unknown	**60 (34)**	4 (50)	11 (28)	45 (34)
***BRAF* status in** **Non-squamous, *n*** (%)				
Mutant	**2 (1)**	0 (0)	0 (0)	2 (2)
Wild type	**51 (29)**	3 (38)	11 (28)	37 (28)
Unknown	**126 (70)**	5 (62)	29 (72)	92 (70)

NOS—not otherwise specified. * at first-diagnosis of metastatic disease.

**Table 3:** Radiotherapy treatment in all metastatic NSCLC patients (*n* = 199) by age group.

Radiotherapy Received	All, *n* (% of Total)	18–30 Years	31–40 Years	41–50 Years
Palliative				
yes	**70 (35)**	4 (44)	12 (27)	54 (37)
no	**129 (65)**	5 (56)	32 (73)	92 (63)
Whole brain				
yes	**44 (22)**	2 (22)	7 (16)	35 (24)
no	**155 (78)**	7 (78)	37 (84)	111 (76)
Stereotactic brain				
yes	**11 (6)**	0 (0)	4 (9)	7 (5)
no	**188 (94)**	9 (100)	40 (91)	139 (95)

**Table 4:** Multivariate analysis of survival in of all metastatic NSCLC patients (*n* = 199; at diagnosis or developed metastatic disease).

Variable	Hazard Ratio	95% Confidence Interval	*p* Value
**Age (yrs)**			
18–30	1.0	--	--
31–40	0.70	0.29–2.12	0.483
41–50	0.60	0.25–1.80	0.305
**Sex**			
Female	1.0	--	--
Male	1.03	0.72–1.48	0.855
**Smoking history**			
Never smoker	0.59	0.34–1.00	0.052
Ex-smoker	1.19	0.72–1.91	0.488
Smoker	1.0	--	--
**Ethnic background**			
White (British, Irish, other)	1.0	--	--
Black (African, Caribbean, British)	0.83	0.53–1.28	0.406
Asian (Asian, British)	0.84	0.32–1.86	0.700
Mixed	1.01	0.16–3.31	0.991
Other	2.89	0.65–9.03	0.105
Number of metastatic sites *, *n* (%)			
1	0.38	0.24–0.62	**<0.001**
2	0.70	0.45–1.10	0.113
>3	1.0	--	--
**Intracranial mets**			
Yes	1.0	--	--
No	1.0	0.69–1.47	0.986
**Histopathology**			
Adenocarcinoma	1.0	--	--
Squamous cell carcinoma	1.69	0.95–2.84	0.060
Large cell carcinoma	2.28	1.92–52.58	**0.004**
NSCLC-NOS	13.02	1.26–3.94	**0.001**
**TKI-option**			
Yes No	0.42	0.24–0.69	**<0.001**
	1.0	--	**--**

NOS—not otherwise specified. Significant *p* values in **bold**. * at first-diagnosis of metastatic disease.

## Data Availability

Data are available upon reasonable request. Deidentified participant data are available upon request from Dr. Alexandros Georgiou. Reuse is permitted where aggregate data will advance research in this field, for example inter-nation or cross-specialty studies of this kind.

## Supplementary Materials

The following supporting information can be downloaded at: https://www.mdpi.com/article/10.3390/cancers14246056/s1, Table S1: Clinical characteristics of all lung cancer patients <50 years (*n* = 301); Table S2: Clinical characteristics of all metastatic (any time point) NSCLC never-smoker patients (*n* = 56); Table S3: Clinical characteristics of all metastatic (any time point) NSCLC patients diagnosed before (*n* = 100) and from (*n* = 99) 1 September 2015.
