# Supplementary material for: Non-Small Cell Lung Cancer (NSCLC) in Young Adults, Age < 50, Is Associated with Late Stage at Presentation and a Very Poor Prognosis in Patients That Do Not Have a Targeted Therapy Option: A Real-World Study
Source: Cancers (Basel). 2022 Dec 9;14(24):6056. doi: 10.3390/cancers14246056 (PMC9776398; doi:10.3390/cancers14246056)
Supplement: Supplementary file 1 [file cancers-14-06056-s001.zip › cancers-2016150-supplementary.pdf]

Supplementary table 1. Clinical characteristics of all lung cancer patients <50 years (n=301).

**Clinical characteristic**	**Total n = 301**
**Age at diagnosis, n (%)**	
18-30	16 (5)
31-40	61 (20)
41-50	224 (74)
mean (yrs)	44
median (yrs)	45
range (yrs)	21-50
**Gender, n (%)**	
Female	146 (49)
Male	155 (51)
**Smoking history, n (%)**	
Never smoker	72 (24)
Ex-smoker	62 (21)
Smoker	114 (38)
Unknown	53 (18)
**Ethnic background, n (%)**	
White (British, Irish, other)	177 (59)
Black (African, Caribbean, British)	52 (17)
Asian (Asian, British)	13 (4)
Mixed	5 (2)
other	9 (3)
not stated	45 (15)
**Stage at diagnosis, n (%)**	
I	45 (15)
II	17 (6)
III	59 (20)
IV	180 (60)
**Histopathology, n (%)**	
Adenocarcinoma	191 (63)
Squamous cell carcinoma	33 (11)
Large cell carcinoma	3 (1)
NSCLC-NOS	21 (7)
Small cell carcinoma	27 (9)
Typical carcinoid	21 (7)
Atypical carcinoid	5 (2)

Supplementary table 2. Clinical characteristics of all metastatic (any time point) NSCLC never-smoker patients (n=56) by age group.

**Clinical characteristic**	**All, n (% of total)**	**18-30 years**	**31-40 years**	**41-50 years**
**Age at diagnosis, n (%)**	**56 (100)**	5 (9)	20 (36)	31 (55)
mean (yrs)	**41**			
median (yrs)	**42**			
range (yrs)	**26-50**			
**Gender, n (%)**				
Female	**41 (73)**	4 (80)	13 (65)	24 (78)
Male	**15 (27)**	1 (20)	7 (35)	7 (23)
**Smoking history, n (%)**				
Never smoker	**56 (100)**	5 (100)	20 (100)	31 (100)
**Ethnic background, n (%)**				
White (British, Irish, other)	**25 (45)**	5 (100)	7 (35)	13 (42)
Black (African, Caribbean, British)	**15 (27)**	0 (0)	4 (20)	11 (35)
Asian (Asian, British)	**4 (7)**	0 (0)	4 (20)	0 (0)
Mixed	**3 (5)**	0 (0)	1 (5)	0 (0)
other	**3 (5)**	0 (0)	1 (5)	2 (6)
not stated	**8 (14)**	0 (0)	3 (15)	5 (16)
**Stage at diagnosis, n (%)**				
I	**0 (0)**	0 (0)	0 (0)	0 (0)
II	**0 (0)**	0 (0)	0 (0)	0 (0)
III	**6 (11)**	1 (20)	1 (5)	4 (13)
IV	**50 (89)**	4 (80)	19 (95)	27 (87)
**Performance status at diagnosis, n (%)**				
0	**13 (23)**	0 (0)	5 (25)	8 (26)
1	**34 (61)**	5 (100)	12 (60)	17 (55)
2	**5 (9)**	0 (0)	2 (10)	3 (10)
3	**1 (2)**	0 (0)	0 (0)	1 (3)
4	**0 (0)**	0 (0)	0 (0)	0 (0)
unknown	**3 (5)**	0 (0)	1 (5)	2 (6)
**Histopathology, n (%)**				
Adenocarcinoma	**48 (86)**	4 (80)	17 (85)	27 (87)
Squamous cell carcinoma	**4 (7)**	1 (20)	1 (5)	2 (6)
Large cell carcinoma	**1 (2)**	0 (0)	1 (5)	0 (0)
NSCLC-NOS	**3 (5)**	0 (0)	1 (5)	2 (6)
**PD-L1 score, n (%)**				
<1	**8 (14)**	0 (0)	4 (20)	4 (13)
1-49	**7 (13)**	0 (0)	3 (15)	4 (13)
≥50	**15 (27)**	3 (60)	5 (25)	7 (23)
unknown	**26 (46)**	2 (40)	8 (40)	16 (52)
***EGFR* status in non-squamous, n (%)**				

Mutant	**20 (36)**	1 (20)	7 (35)	12 (39)
Wild type	**33 (59)**	3 (60)	12 (60)	18 (58)
unknown	**3 (5)**	1 (20)	1 (5)	1 (3)
***ALK* status in non-squamous, n (%)**				
Rearranged	**11 (20)**	2 (40)	6 (30)	3 (10)
Not rearranged	**33 (59)**	2 (40)	11 (55)	20 (65)
unknown	**12 (21)**	1 (20)	3 (15)	8 (26)
***ROS1* status in non-squamous, n (%)**				
Rearranged	**5 (9)**	1 (20)	3 (15)	1 (3)
Not rearranged	**32 (57)**	2 (40)	12 (60)	18 (58)
unknown	**19 (34)**	2 (40)	5 (25)	12 (39)
***KRAS* status in non-squamous, n (%)**				
Mutant	**2 (4)**	0 (0)	1 (5)	1 (3)
Wild type	**36 (64)**	3 (60)	12 (60)	21 (68)
unknown	**18 (32)**	2 (40)	7 (35)	9 (29)
***BRAF* status in non-squamous, n (%)**				
Mutation	**0 (0)**	0 (0)	0 (0)	0 (0)
Wild type	**24 (43)**	3 (60)	6 (30)	15 (48)
unknown	**32 (57)**	2 (40)	14 (70)	16 (52)

Supplementary table 3. Clinical characteristics of all metastatic (any time point) NSCLC patients diagnosed before (n=100) and from (n=99) 1st September 2015.

**Clinical characteristic**	**All, n (% of total)**	**Pre-Sept 2015**	**Post-Sept 2015**
**Age at diagnosis, n (%)**	**199 (100)**	100 (50)	99 (50)
mean (yrs)	**44**	44	43
median (yrs)	**45**	45	45
range (yrs)	**26-50**	27-50	26-50
**Gender, n (%)**			
Female	**97 (49)**	44 (44)	53 (54)
Male	**102 (51)**	56 (56)	46 (46)
**Smoking history, n (%)**			
Never smoker	**56 (28)**	21 (21)	35 (35)
Ex smoker	**38 (19)**	19 (19)	19 (19)
Smoker	**75 (38)**	35 (35)	40 (40)
Unknown	**30 (15)**	25 (25)	5 (5)
**Ethnic background, n (%)**			
White (British, Irish, other)	**109 (55)**	55 (55)	54 (55)
Black (African, Caribbean, British)	**41 (21)**	26 (26)	15 (15)
Asian (Asian, British)	**10 (5)**	8 (8)	2 (2)
Mixed	**3 (2)**	0 (0)	3 (3)
other	**4 (2)**	2 (2)	2 (2)
not stated	**32 (16)**	9 (9)	23 (23)
**Stage at diagnosis, n (%)**			
I	**6 (3)**	2 (2)	4 (4)
II	**2 (1)**	2 (2)	0 (0)
III	**25 (13)**	16 (16)	9 (9)
IV	**166 (83)**	80 (80)	86 (87)
**Performance status at diagnosis, n (%)**			
0	**39 (20)**	19 (19)	20 (20)
1	**101 (51)**	51 (51)	50 (51)
2	**18 (9)**	9 (9)	9 (9)
3	**12 (6)**	5 (5)	7 (7)
4	**4 (2)**	3 (3)	1 (1)
unknown	**25 (13)**	13 (13)	12 (12)
**Histopathology, n (%)**			
Adenocarcinoma	**159 (80)**	80 (80)	79 (80)
Squamous cell carcinoma	**20 (10)**	9 (9)	11 (11)
Large cell carcinoma	**2 (1)**	1 (1)	1 (1)
NSCLC-NOS	**18 (9)**	10 (10)	8 (8)

**PD-L1 score, n (%)**			
<1	**32 (16)**	3 (3)	29 (29)
1-49	**18 (9)**	2 (2)	16 (16)
≥50	**32 (16)**	1 (1)	31 (31)
unknown	**117 (59)**	94 (94)	23 (23)
***EGFR* status in non-squamous, n (%)**			
Mutant	**34 (19)**	16 (16)	19 (19)
Wild type	**123 (69)**	63 (63)	66 (67)
unknown	**22 (12)**	21 (21)	14 (14)
***ALK* status in non-squamous, n (%)**			
Rearranged	**18 (10)**	3 (3)	15 (15)
Not rearranged	**100 (56)**	43 (43)	64 (65)
unknown	**61 (34)**	54 (54)	20 (20)
***ROS1* status in non-squamous, n (%)**			
Rearranged	**8 (4)**	4 (4)	5 (5)
Not rearranged	**94 (53)**	33 (33)	66 (67)
unknown	**77 (43)**	63 (63)	28 (28)
***KRAS* status in non-squamous, n (%)**			
Mutant	**22 (12)**	9 (9)	13 (13)
Wild type	**97 (54)**	41 (41)	60 (61)
unknown	**60 (34)**	50 (50)	26 (26)
***BRAF* status in non-squamous, n (%)**			
Mutation	**2 (1)**	0 (0)	2 (2)
Wild type	**51 (29)**	5 (5)	49 (49)
unknown	**126 (70)**	95 (95)	48 (48)
